# Operational Approach to the Lancet 2025 Obesity Framework in Mexican Adults: Prevalence and Clinical Characteristics

**DOI:** 10.3390/nu18152582

**Published:** 2026-08-06

**Authors:** Daniela L. C. Delgado-Lara, Elizabeth Hernández-Castellanos, Diana J. Mejía-Barajas, Haru C. B. Angel-González, Sofía F. Angulo-Camacho, Omar Graciano-Machuca, Valeria Diaz-Rizo, Liliana Iñiguez-Gutiérrez, Teresita J. Hernández-Flores

**Affiliations:** 1 Departamento de Fundamentos Biológicos, Facultad de Medicina, Universidad Autónoma de Guadalajara, Zapopan 45129, Jalisco, Mexico; daniela.delgado@edu.uag.mx; 2 School of Dietetics and Human Nutrition, McGill University, Sainte-Anne-de-Bellevue, QC H9X 3V9, Canada; elizabeth.hernandezcastellanos@mail.mcgill.ca; 3 Licenciatura en Nutrición, Centro Universitario de Ciencias de la Salud, Universidad de Guadalajara, Guadalajara 44340, Jalisco, Mexico; 4 Centro de Investigación en Procesamiento Digital de Señales, Centro Universitario de los Valles, Universidad de Guadalajara, Ameca 46600, Jalisco, Mexico; omargmachuca@academicos.udg.mx; 5 Instituto de Investigación de Recursos Humanos en Salud, Departamento de Disciplinas Filosófico, Metodológicas e Instrumentales, Centro Universitario de Ciencias de la Salud, Universidad de Guadalajara, Guadalajara 44340, Jalisco, Mexico; valeria_dr@academicos.udg.mx; 6 Departamento de Ciencias de la Salud, Centro Universitario de los Valles, Universidad de Guadalajara, Ameca 46600, Jalisco, Mexico; 7 Departamento de Disciplinas Especializadas I, Decanato de Ciencias de la Salud, Universidad Autónoma de Guadalajara, Zapopan 45129, Jalisco, Mexico; 8 Departamento de Alimentación y Nutrición, Centro Universitario de Ciencias de la Salud, Universidad de Guadalajara, Guadalajara 44340, Jalisco, Mexico

**Keywords:** excess adiposity, preclinical obesity, clinical obesity, waist-to-height ratio, cardiometabolic risk, obesity-related dysfunction, national health survey

## Abstract

**Background/Objectives:** The 2025 *Lancet Diabetes & Endocrinology* Commission proposed a framework that distinguishes preclinical from clinical obesity based on confirmed excess adiposity and evidence of obesity-related organ dysfunction. We estimated the prevalence and clinical characteristics of preclinical and clinical obesity in Mexican adults and quantified their discordance with conventional body mass index (BMI) categories. **Methods:** Cross-sectional analysis of 5014 adults aged ≥20 years (weighted population 77,915,402) from the 2023 Mexican National Health and Nutrition Survey (ENSANUT). Excess adiposity was confirmed by a BMI ≥ 30 kg/m^2^ plus one altered measure of central adiposity, by two altered measures irrespective of BMI, or by a BMI ≥ 40 kg/m^2^ alone. Obesity-related dysfunction was ascertained from available cardiovascular, metabolic, and functional indicators. All analyses accounted for the complex sampling design. **Results:** Preclinical obesity affected 30.9% of adults and clinical obesity 19.6%. Among adults with clinical obesity, 4.9% had a normal BMI and 18.3% were overweight so that 23.2% fell below the conventional 30 kg/m^2^ threshold. Cardiovascular dysfunction was the most frequent qualifying domain (78.1%), followed by limitations in activities of daily living (27.6%) and metabolic dysfunction (6.7%). **Conclusions:** BMI alone underestimates obesity-related clinical severity and misses a substantial share of adults with organ dysfunction, supporting the integration of central adiposity measures and assessment of obesity-related dysfunction into routine obesity surveillance.

## 1. Introduction

Obesity is a major public health challenge worldwide, and its prevalence has increased steadily in recent decades. Approximately 16% of adults worldwide currently have obesity, representing some 890 million people [1,2]. The World Obesity Federation projects a 115% increase in obesity prevalence by 2030 relative to 2010 [3].

Because body mass index (BMI) is simple, inexpensive, and easy to implement, it has been used as a screening tool for overweight and obesity in epidemiological settings and as a diagnostic criterion in clinical practice [4]. Despite its widespread use, BMI has important limitations: It does not capture variation in body composition across sex and ethnic groups; it has limited value for individual risk assessment and may under- or overestimate risk for certain body profiles; it does not distinguish fat mass from lean mass and may misclassify individuals with high muscle mass; and it does not reflect fat distribution, despite the clinical relevance of visceral adiposity to metabolic risk [5]. It has therefore been proposed that BMI be considered alongside other anthropometric and clinical indicators when a clinical diagnosis is made [6].

To address these limitations, *The Lancet Diabetes & Endocrinology* Commission (2025) proposed a diagnostic framework that reconceptualizes obesity as excess adiposity and operationalizes its diagnosis through a two-step algorithm [7].

The first step of the algorithm establishes whether excess adiposity is present. Because BMI has well-documented limitations as a marker of adiposity, it serves only as a screening entry point, applied with sex- and ethnicity-specific thresholds, and requires corroboration rather than being used in isolation. Excess adiposity is confirmed when any one of the following criteria is met: (i) a BMI in the obesity range together with at least one measure of central adiposity above its sex-specific cutoff (waist circumference, waist-to-hip ratio, or waist-to-height ratio); (ii) two such measures above their cutoffs, regardless of BMI; or (iii) direct assessment of body fat, for example by DXA or bioelectrical impedance. In individuals with a BMI ≥ 40 kg/m^2^, excess adiposity can be presumed pragmatically without further confirmation [7]. Pathway (ii) is particularly relevant to the present analysis because it allows obesity status to be confirmed in individuals whose BMI falls near but below the conventional obesity threshold: two anthropometric measures consistent with excess adiposity are sufficient, regardless of BMI. The Commission further notes that BMI can underestimate adiposity, especially at values around the traditional thresholds, and that individuals presenting with typical manifestations of clinical obesity may have a BMI below the recommended cutoffs; such individuals should be assessed for excess adiposity using alternative measurements [7].

The second step establishes whether excess adiposity has affected the function of organs, tissues, or the individual as a whole. Individuals with confirmed excess adiposity in whom the function of other tissues and organs is preserved, with no obesity-related limitation of activities of daily living, are classified as having preclinical obesity. This category denotes a state of risk rather than established disease: preclinical obesity confers an increased likelihood of progressing to clinical obesity and of developing other noncommunicable diseases. Individuals in whom excess adiposity is accompanied by signs, symptoms, or diagnostic-test evidence of organ or tissue dysfunction, or by substantial, age-adjusted limitation of activities of daily living attributable to obesity, are classified as having clinical obesity, which the Commission defines as a chronic, systemic illness in its own right [7].

For adults, the Commission specifies 18 diagnostic criteria spanning the central nervous system, upper airways, respiratory system, cardiovascular system, metabolism, liver, kidney, urinary tract, reproductive system, musculoskeletal system, and lymphatic system, together with substantial limitation of activities of daily living. Most of these criteria require clinical, laboratory, or imaging assessments that population-based surveys do not collect. Two features of the criteria are therefore decisive for applying the framework to survey data, and they operate in opposite directions. First, elevated arterial blood pressure is itself a listed cardiovascular criterion (alongside heart failure with reduced or preserved ejection fraction, chronic or recurrent atrial fibrillation, pulmonary artery hypertension, and recurrent deep vein thrombosis or pulmonary thromboembolic disease), so that a measurement routinely obtained in national surveys is sufficient to establish cardiovascular dysfunction. Second, the metabolic criterion is defined as a cluster of hyperglycemia, hypertriglyceridemia, and low HDL cholesterol; an isolated metabolic abnormality therefore does not satisfy it, and, in the absence of any other qualifying criterion, the individual is classified as having preclinical obesity. Beyond these two features, all criteria are met only when other obvious causes of the dysfunction have been excluded, and the coexistence of other obesity-related diseases does not, by itself, count as obesity-related dysfunction; obesity is thus diagnosed as an independent entity rather than through co-occurring diseases [7].

Since the framework was published in January 2025, a small number of studies have applied it to national surveys and cohorts, with operational approaches that vary according to the variables available in each data source. One analysis confirmed excess adiposity but did not assess the clinical component and therefore could not separate preclinical from clinical obesity [8]. Others ascertained organ dysfunction from ICD-10 codes or from recorded diagnoses of hypertension or diabetes rather than from the signs of organ dysfunction that the Commission specifies [9,10]. Only one study has applied both steps and corroborated the resulting classification against prospectively recorded events, in two Chinese cohorts [11]. Whether the framework can be applied to a nationally representative survey using measured signs rather than diagnostic labels, therefore, remains largely untested.

The Commission’s reconceptualization of obesity has direct implications for population-based research, particularly in countries such as Mexico, where obesity and obesity-related diseases are highly prevalent. In the most recent national estimates, 36.9% of Mexican adults had obesity, and a further 38.3% had overweight by BMI, while abdominal obesity, reported as an independent indicator, reached 81.0% [12]. The framework, however, combines these measures rather than treating them separately. We therefore applied the Commission’s two-step algorithm to individual-level data from the 2023 Mexican National Health and Nutrition Survey (ENSANUT), operationalizing each criterion with the variables the survey provides and ascertaining organ dysfunction from measured signs rather than from recorded diagnoses of established disease. Our aims were to estimate the prevalence and clinical characteristics of preclinical and clinical obesity in Mexican adults, and to quantify the discordance between the resulting classification and conventional BMI categories.

## 2. Materials and Methods

### 2.1. Design and Population

We conducted a cross-sectional analysis of the ENSANUT 2023, a survey that provides nationally representative estimates stratified by urban and rural area and by geographic region [13]. Individual-level records from three ENSANUT datasets (anthropometry and blood pressure, adult health, and physical activity) were linked by participant identifier.

Two number systems are used throughout and are not interchangeable: unweighted counts (*n*) refer to survey records and describe eligibility, exclusions, and domain coverage, whereas weighted counts (*N*) estimate the corresponding Mexican adult population and underlie every prevalence estimate reported.

**Figure 1:**
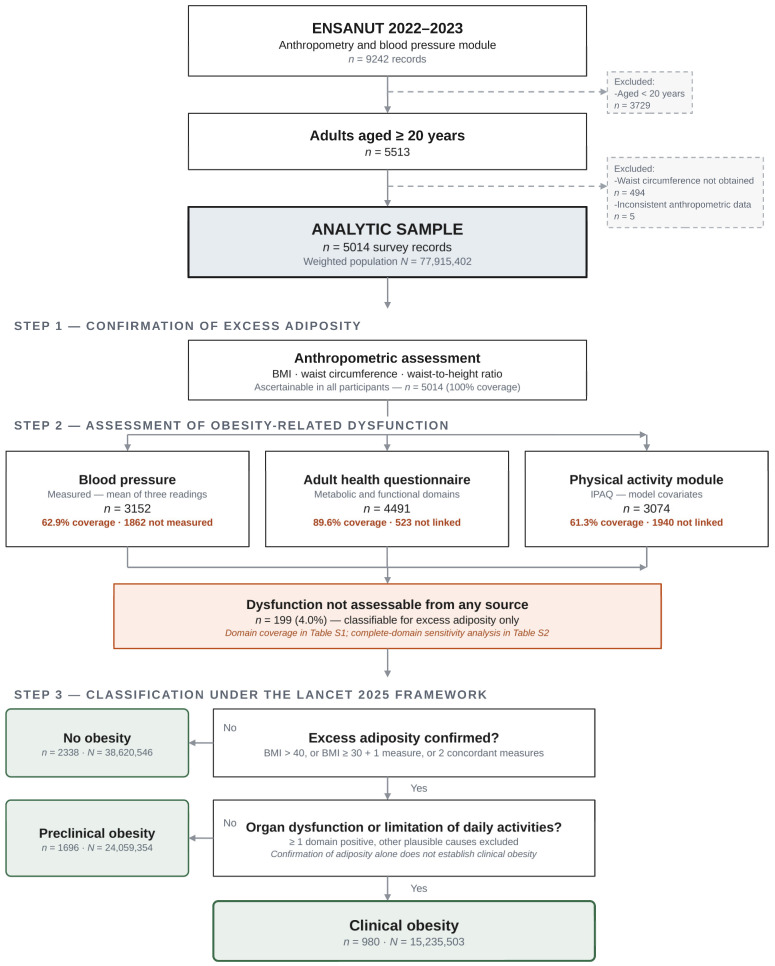
Flow diagram of participant selection from the Mexican National Health and Nutrition Survey (ENSANUT) 2023. All counts are unweighted numbers of survey records (*n*); weighted population estimates (*N*) are reported in the text and in Table 1, Table 2, Table 3, Table 4 and Table 5. Solid arrows indicate sequential flow through the selection and classification steps; dashed arrows indicate exclusion from the analytic sample; and “Yes”/”No” arrows in Step 3 indicate whether the classification criterion was met. Five records with internally inconsistent anthropometric measurements (body weight, height, and BMI mutually incompatible) were excluded from the analytic sample, yielding *n* = 5014. Excess adiposity was ascertainable in every participant, whereas assessment of obesity-related dysfunction was constrained by module-specific coverage. A participant with confirmed excess adiposity could therefore be classified as having preclinical obesity solely because no dysfunction domain was assessed; coverage percentages denote the proportion of the 5014 analytic-sample records for which the corresponding module was available. Sarcopenia identified by the SARC-F scale (*n* = 165) was excluded from the functional domain only; these participants remain in the analytic sample for all other analyses. BMI, body mass index; ENSANUT, Encuesta Nacional de Salud y Nutrición; IPAQ, International Physical Activity Questionnaire.

Of 9242 records in the anthropometry module, 3729 were excluded for age under 20 years, 494 for lacking a valid waist circumference, and 5 for mutually incompatible values of body weight, height, and BMI, indicative of data-entry error. The analytic sample therefore comprised *n* = 5014 adults aged ≥ 20 years, representing a weighted population of *N* = 77.9 million Mexican adults (Figure 1).

Excess adiposity was ascertainable in all 5014 participants, whereas assessment of obesity-related dysfunction was constrained by module-specific coverage. Blood pressure was measured in 3152 participants (62.9%), and the adult health questionnaire was available for 4491 (89.6%); because the cardiovascular domain could be established from either source, it was classifiable in 4809 participants (95.9%). The metabolic domain was classifiable in 4491 participants (89.6%) and the functional domain in 4497 (89.7%). In 199 participants (4.1%), no dysfunction domain could be assessed, and classification for these individuals was therefore limited to confirmed excess adiposity. Domain-specific coverage is detailed in Table S1, and a complete-domain sensitivity analysis is in Table S2.

Within the older-adult functional subgroup, 165 participants with sarcopenia identified by the SARC-F scale were excluded from that domain only, to avoid misattributing reduced mobility to obesity when it reflected sarcopenia; they remain in the analytic sample for all other analyses.

### 2.2. Identification of Excess Adiposity

The Commission [7] does not prescribe how its criteria should be operationalized with existing population-survey data. As in previous applications of this framework to nationally representative surveys [10,14,15,16,17], each diagnostic criterion was therefore mapped to the closest available ENSANUT 2023 indicator; this mapping and the thresholds applied are presented in Supplementary Table S1.

Anthropometric measurements were collected by trained personnel using standardized procedures. Repeated measurements of height, weight, and waist circumference were averaged to obtain the final analytic values, from which BMI and waist-to-height ratio (WHtR) were calculated. For older adults whose standing height could not be measured, height was estimated from knee height. Adults aged 20–59 years were classified using conventional BMI cutoff points, and those aged ≥60 years using age-specific cutoff points [18,19,20].

Excess adiposity was confirmed when a participant met one of the following criteria: (i) BMI ≥ 40 kg/m^2^; (ii) BMI ≥ 30 kg/m^2^ together with at least one altered body-size measure, defined as a waist circumference ≥ 102 cm in men or ≥88 cm in women, or a WHtR ≥ 0.56 in men or ≥0.61 in women; or (iii) both altered body-size measures simultaneously, regardless of BMI [21]. The WHtR was rounded to two decimal places before the cutoff was applied. These WHtR cutoffs are the sex-specific thresholds derived for the general adult population in the source study [21].

Criterion (i) confirmed excess adiposity but did not by itself establish clinical obesity. Participants with a BMI ≥ 40 kg/m^2^ were classified as having clinical obesity only when organ dysfunction or functional limitation was also documented, and as having preclinical obesity otherwise.

### 2.3. Definition of Obesity-Related Dysfunction and Functional Limitation

Consistent with the Commission’s [7] distinction between a diagnostic sign and a coexisting disease (see Introduction), each domain was defined by measured signs rather than by diagnostic labels: a prior diagnosis neither established nor precluded the presence of dysfunction. Prevalent diagnoses of diabetes, hypertension, and cardiovascular and renal disease are reported separately as descriptive clinical characteristics. Domain coverage is reported in Table S1.

Metabolic dysfunction required the simultaneous presence of hyperglycemia, hypertriglyceridemia, and low HDL cholesterol. Hyperglycemia was defined as self-reported prediabetes, impaired fasting glucose, or impaired capillary glucose; a self-reported prior diagnosis of type 2 diabetes was not included in this definition and was evaluated separately in a sensitivity analysis (Table S2). Self-reported diagnoses of these conditions have shown acceptable positive predictive value when validated against measured values [22]. HDL cholesterol was not measured in the ENSANUT 2023; hypercholesterolemia was therefore substituted for the low-HDL component. Because total and HDL cholesterol are not interchangeable markers, this substitution alters the composition of the metabolic domain rather than approximating the original criterion, and the resulting prevalence of metabolic dysfunction is not directly comparable with applications of the framework that include HDL cholesterol.

Cardiovascular dysfunction was ascertained from elevated blood pressure, defined as a systolic pressure ≥120 mmHg or a diastolic pressure ≥ 80 mmHg following the 2025 AHA/ACC blood-pressure categories, in which normal blood pressure requires both values to fall below these thresholds [23]. Blood pressure was recorded as the mean of three measurements taken by trained personnel and was available for 3152 of the 5014 participants (62.9%); when measured values were unavailable, cardiovascular dysfunction was ascertained from a self-reported history of having been told, during a health consultation, that blood pressure was elevated.

Limitations in daily life were assessed with the Katz Index (score ≥ 3), the Lawton–Brody scale (score > 0), or a fall-risk indicator of at least two falls per year [24]; these instruments were administered only to older adults. Sarcopenia was identified with the SARC-F scale [25], and functional limitation was attributed to obesity only in the absence of SARC-F-identified sarcopenia.

Clinical obesity requires a single documented dysfunction, whereas preclinical obesity requires the absence of dysfunction across all assessable domains. Because domain coverage in the ENSANUT 2023 is incomplete (Section 2.1, Figure 1), misclassification arising from incomplete coverage is directional rather than random: it can only lead to under-ascertainment of clinical obesity and to corresponding over-assignment to preclinical obesity. The estimates of clinical obesity reported here should therefore be interpreted as conservative lower bounds. Accordingly, participants with confirmed excess adiposity and no detected dysfunction were classified as having preclinical obesity, a category that reflects the absence of detected dysfunction across the domains assessed rather than verified preservation of function in every organ system.

### 2.4. Statistical Analysis

All analyses accounted for the complex survey design of the ENSANUT 2023, including sampling weights, stratification, and clustering. Continuous variables were summarized as weighted means with standard errors and 95% confidence intervals (CIs), and categorical variables were summarized as weighted prevalences with 95% CIs. Sex-stratified and age-stratified comparisons used adjusted Wald tests for continuous variables and second-order Rao–Scott-corrected chi-square tests for categorical variables; the distribution of qualifying dysfunction domains within each age stratum was described using the same weighted methods (Table S3). Complex-sample multinomial logistic regression was used to examine factors associated with the Lancet 2025 categories, using the without-obesity group as the reference. The models were progressively adjusted for age, sex, smoking, episodic binge drinking, CESD-7 score, physical activity (METs), and sleep duration. Definitional variables were excluded from the regression model. Model 3, based on a subset with complete information, was interpreted as a complete-case sensitivity analysis. A sensitivity analysis extended the metabolic dysfunction domain to include a previously reported diagnosis of type 2 diabetes, in addition to the self-reported hyperglycemia criteria used in the main analysis (Section 2.3, Table S2). Analyses were performed using IBM SPSS Statistics for Mac, Version 32.0 (IBM Corp., Armonk, NY, USA), applying the Complex Samples module to account for the survey’s stratified, clustered design and sampling weights. A *p*-value less than 0.05 was considered statistically significant.

## 3. Results

### 3.1. Sociodemographic, Lifestyle, and Anthropometric Characteristics

The weighted study population represented 77,915,402 adults, with a similar sex distribution and no significant age difference between women and men (43.67 vs. 43.53 years; *p* = 0.808). Sex differences were observed in lifestyle, behavioral, and mental health factors. Men more frequently reported current or former smoking (51.84% vs. 23.07%; *p* < 0.001), whereas the prevalence of episodic binge drinking did not differ significantly between sexes. Women reported poorer mental health, with higher CESD-7 scores and a higher prevalence of depressive symptoms than men. Men also had higher physical activity levels, both in mean METs and in the proportion classified as vigorously active (Table 1).

**Table 1:** Sociodemographic, lifestyle, and clinical characteristics of Mexican adults 20 years and older, by sex, ENSANUT 2023 (*n* = 5014).

Variable	Total *N* = 77,915,402	Female *N* = 38,870,096	Male *N* = 39,045,306	*p* Value
Age (years)	43.60 ± 0.348 [42.92–44.28]	43.66 ± 0.388 [42.90–44.43]	43.53 ± 0.494 [42.56–44.50]	0.808
**Lifestyle factors**				
Current/former smoker (yes)	37.09 [34.89–39.28]	23.07 [20.89–25.25]	51.83 [48.90–54.77]	<0.001
Episodic binge drinking (yes)	46.40 [43.20–49.60]	42.79 [37.89–47.69]	48.49 [43.63–53.34]	0.141
**Mental health and sleep**				
CESD-7 depression (score)	3.83 ± 0.076 [3.68–3.98]	4.48 ± 0.128 [4.23–4.73]	3.15 ± 0.076 [3.00–3.30]	<0.001
Depression (yes)	24.79 [23.22–26.36]	31.80 [29.16–34.44]	17.42 [15.46–19.39]	<0.001
Sleep (hours)	7.08 ± 0.029 [7.02–7.13]	7.11 ± 0.036 [7.04–7.19]	7.04 ± 0.035 [6.97–7.11]	0.062
Sufficient sleep (yes)	31.57 [29.27–33.86]	30.41 [27.52–33.30]	32.74 [29.39–36.10]	0.284
**Physical activity**				
IPAQ physical activity (METs)	6859.2 ± 237.00 [6394.7–7323.8]	4999.3 ± 266.98 [4476.0–5522.6]	8748.7 ± 392.88 [7978.6–9518.7]	<0.001
**IPAQ physical activity classification**				
Low	25.14 [23.26–27.02]	28.52 [25.89–31.15]	21.70 [19.04–24.36]	<0.001
Moderate	22.58 [20.63–24.54]	24.55 [22.11–26.99]	20.58 [17.59–23.58]	
Vigorous	52.28 [50.21–54.34]	46.93 [44.19–49.67]	57.72 [54.81–60.62]	
**General anthropometric indicators**				
BMI (kg/m^2^)	29.14 ± 0.096 [28.96–29.33]	29.57 ± 0.152 [29.27–29.87]	28.72 ± 0.133 [28.46–28.98]	<0.001
**BMI category**				
Underweight	1.88 [1.52–2.25]	1.15 [0.77–1.54]	2.61 [1.82–3.41]	<0.001
Normal weight	24.58 [23.08–26.09]	23.72 [21.84–25.59]	25.45 [23.34–27.56]	
Overweight	35.01 [33.32–36.69]	33.62 [31.03–36.20]	36.39 [34.23–38.55]	
Obesity	38.52 [36.87–40.18]	41.51 [38.86–44.17]	35.55 [33.28–37.82]	
**Central adiposity and blood pressure indicators**				
WHtR (score)	0.61 ± 0.002 [0.60–0.61]	0.62 ± 0.003 [0.62–0.63]	0.59 ± 0.002 [0.59–0.60]	<0.001
Elevated WHtR (yes)	62.42 [60.59–64.26]	54.96 [51.98–57.94]	69.85 [67.79–71.92]	<0.001
WC (cm)	97.26 ± 0.269 [96.73–97.79]	95.49 ± 0.345 [94.82–96.17]	99.02 ± 0.331 [98.37–99.67]	<0.001
Elevated WC (yes)	54.44 [52.65–56.23]	68.79 [66.61–70.97]	40.15 [37.62–42.68]	<0.001
SBP (mmHg)	121.63 ± 0.431 [120.79–122.47]	116.42 ± 0.526 [115.39–117.45]	126.62 ± 0.588 [125.47–127.78]	<0.001
Elevated SBP (yes)	46.89 [44.60–49.18]	35.54 [33.08–38.01]	57.77 [54.80–60.74]	<0.001
DBP (mmHg)	74.25 ± 0.254 [73.75–74.75]	72.22 ± 0.319 [71.60–72.85]	76.20 ± 0.328 [75.55–76.84]	<0.001
Elevated DBP (yes)	27.86 [25.71–30.01]	22.52 [20.08–24.97]	32.98 [30.03–35.92]	<0.001
**Previous medical diagnoses**				
Renal dysfunction	20.91 [19.62–22.21]	27.27 [25.27–29.28]	14.22 [12.54–15.90]	<0.001
Type 2 diabetes	10.52 [9.52–11.52]	11.16 [10.01–12.32]	9.85 [8.34–11.36]	0.158
Hypertension	18.00 [16.58–19.41]	19.63 [17.73–21.54]	16.28 [14.36–18.20]	0.012
Cardiovascular disease	4.93 [4.00–5.86]	4.58 [3.74–5.42]	5.30 [3.80–6.80]	0.347
**Components used to operationalize the Lancet 2025 obesity criteria**				
Excess adiposity ^a^	50.43 [48.63–52.23]	57.07 [54.25–59.89]	43.82 [41.49–46.16]	<0.001
Metabolic dysfunction ^b^	1.34 [0.92–1.76]	1.43 [0.99–1.86]	1.24 [0.54–1.95]	0.670
Including non-obesity-related dysfunction	12.33 [11.18–13.47]	12.99 [11.68–14.31]	11.63 [9.88–13.37]	0.201
Cardiovascular dysfunction ^c^	15.96 [14.71–17.21]	13.86 [12.15–15.56]	18.07 [16.25–19.89]	0.001
Including non-obesity-related dysfunction	47.12 [45.12–49.13]	40.91 [38.26–43.56]	53.36 [51.01–55.70]	<0.001
Limitation in activities of daily living ^d^	5.53 [4.78–6.29]	7.07 [5.82–8.31]	3.93 [3.31–4.54]	<0.001

Weighted means ± SE [95% CI] (continuous) or prevalence, % [95% CI] (categorical); accounts for the complex survey design. *p* values: adjusted Wald tests (continuous), second-order Rao–Scott chi-square tests (categorical). ^a^ Operationalized for *The Lancet* 2025 criteria. ^b,c,d^ Obesity-related dysfunction only; indented row adds non-obesity-related cases. Abbreviations: BMI, body mass index; CESD-7, seven-item Center for Epidemiologic Studies Depression scale; DBP, diastolic blood pressure; ENSANUT, Encuesta Nacional de Salud y Nutrición; IPAQ, International Physical Activity Questionnaire; METs, metabolic equivalents of task; SBP, systolic blood pressure; WC, waist circumference; WHtR, waist-to-height ratio.

Excess body weight was highly prevalent, with a mean BMI of 29.14 kg/m^2^, and 73.53% of adults were classified as overweight or obese. Women had a higher mean BMI than men (29.57 vs. 28.72 kg/m^2^; *p* < 0.001) and a higher prevalence of obesity (41.51% vs. 35.55%; *p* < 0.001), whereas overweight was more frequent in men. Women also showed higher central adiposity, with a higher mean WHtR and a greater prevalence of elevated waist circumference, whereas men had higher systolic and diastolic blood pressure values and a greater prevalence of elevated blood pressure (all *p* < 0.001) (Table 1). Regarding previous diagnoses, women more frequently reported renal dysfunction (*p* < 0.001) and hypertension (*p* = 0.012), whereas the prevalence of diabetes and cardiovascular disease did not differ significantly between sexes.

Among the components used to operationalize the Lancet 2025 criteria, excess adiposity was present in 50.43% of the population, with a higher prevalence in women than in men (57.07% vs. 43.82%; *p* < 0.001). Metabolic dysfunction did not differ by sex, whereas cardiovascular dysfunction was more frequent in men, and limitations in activities of daily living were more frequent in women (Table 1).

### 3.2. Distribution of The Lancet 2025 Obesity Categories

**Figure 2:**
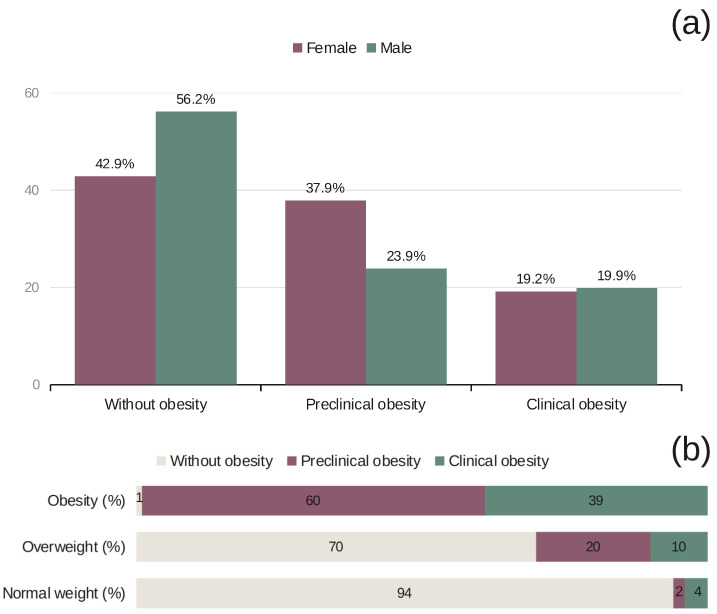
Distribution of *The Lancet* 2025 obesity categories among Mexican adults from ENSANUT 2023. (**a**) Distribution of obesity categories within each sex. Bars show the percentage of female and male participants classified as having without obesity, preclinical obesity, or clinical obesity according to *The Lancet Diabetes & Endocrinology* Commission 2025 criteria. Percentages within each sex sum to 100%. (**b**) Distribution of *The Lancet* 2025 obesity categories within each WHO BMI category (normal weight, BMI 18.5–24.9 kg/m^2^, overweight, BMI 25.0–29.9 kg/m^2^, obesity, BMI ≥ 30.0 kg/m^2^). Stacked bars sum to 100% within each BMI category; values are rounded to whole numbers. BMI: body mass index; ENSANUT: Encuesta Nacional de Salud y Nutrición; WHO: World Health Organization.

According to *The Lancet* 2025 criteria, the weighted adult population was classified as without obesity in 49.6% of cases, as having preclinical obesity in 30.9%, and as having clinical obesity in 19.6%. A sex-specific pattern was observed (*p* < 0.001): men were more frequently classified as without obesity, whereas women showed a higher prevalence of preclinical obesity than men. In contrast, clinical obesity showed little sex difference (Figure 2a).

The distribution of *The Lancet* 2025 obesity categories also varied by age (Supplementary Table S3). The ratio of preclinical to clinical obesity was 2.28 among adults aged 20–29 years, peaked at 2.53 in the 30–39-year group, and declined monotonically thereafter, falling to 0.30 among those aged 70 years and older and below 1 in the 60–69-year group; that is, clinical obesity was more prevalent than preclinical obesity among adults aged 60 years and older. This shift was accompanied by a change in the predominant qualifying dysfunction domain: cardiovascular dysfunction accounted for 94.7% of clinical obesity cases in the youngest age group but only 46.7% in the oldest, whereas limitations in activities of daily living rose from 5.8% to 70.2% over the same age range (Table S3).

### 3.3. Anthropometric and Hemodynamic Profile by Category

Adults with clinical obesity showed the least favorable anthropometric and hemodynamic profiles. Age increased progressively across diagnostic categories, from 40.27 years in those without obesity to 43.79 years in preclinical obesity and 51.73 years in clinical obesity (*p* < 0.001). WHtR and waist circumference increased progressively across categories and were highest in clinical obesity, whereas BMI plateaued beyond the BMI-defined obesity threshold, with similar mean values in preclinical and clinical obesity (33.22 vs. 33.13 kg/m^2^) (Table 2).

**Table 2:** Anthropometric and blood pressure characteristics of Mexican adults aged 20 years and older, by *The Lancet* 2025 obesity category, ENSANUT 2023 (*n* = 5014).

Variable	Without Obesity *N* = 38,620,546	Preclinical Obesity *N* = 24,059,354	Clinical Obesity *N* = 15,235,503	*p* Value
Age (years)	40.27 ± 0.489 [39.31–41.22]	43.79 ± 0.320 [43.17–44.42]	51.73 ± 0.679 [50.40–53.06]	<0.001
BMI (kg/m^2^)	25.03 ± 0.075 [24.88–25.18]	33.22 ± 0.147 [32.94–33.51]	33.13 ± 0.155 [32.82–33.43]	<0.001
BMI-defined obesity (%)	0.63 [0.34–1.18]	75.05 [72.50–77.44]	76.89 [74.25–79.34]	<0.001
WHtR	0.54 ± 0.001 [0.54–0.54]	0.67 ± 0.002 [0.67–0.68]	0.68 ± 0.003 [0.67–0.68]	<0.001
Elevated WHtR (%)	26.91 [25.04–28.87]	96.59 [95.56–97.38]	98.50 [95.56–99.50]	<0.001
WC (cm)	87.05 ± 0.206 [86.65–87.46]	106.64 ± 0.345 [105.96–107.32]	108.32 ± 0.479 [107.38–109.26]	<0.001
Elevated WC (%)	11.82 [10.07–13.83]	95.71 [93.82–97.04]	97.29 [95.84–98.25]	<0.001
SBP (mmHg)	117.85 ± 0.709 [116.46–119.24]	118.20 ± 0.862 [116.51–119.89]	132.52 ± 0.698 [131.15–133.89]	<0.001
Elevated SBP (%)	37.92 [34.31–41.67]	28.26 [24.69–32.12]	83.35 [80.55–85.82]	<0.001
DBP (mmHg)	71.79 ± 0.304 [71.19–72.38]	73.12 ± 0.607 [71.93–74.31]	80.22 ± 0.371 [79.50–80.95]	<0.001
Elevated DBP (%)	19.73 [17.53–22.13]	18.86 [15.42–22.85]	52.91 [49.20–56.60]	<0.001

Weighted means ± SE [95% CI] (continuous) or prevalence, % [95% CI], logit method (categorical); accounts for the complex survey design. *p*-Values: adjusted Wald tests (continuous), second-order Rao–Scott chi-square tests (categorical). Abbreviations: BMI, body mass index; DBP, diastolic blood pressure; ENSANUT, Encuesta Nacional de Salud y Nutrición; SBP, systolic blood pressure; WC, waist circumference; WHtR, waist-to-height ratio.

Hemodynamic impairment was also most pronounced in clinical obesity; mean SBP and DBP, as well as the prevalence of elevated SBP and DBP, were markedly higher in this group than in the preclinical or without-obesity groups (all *p* < 0.001). SBP and DBP values were relatively similar between participants without obesity and those with preclinical obesity, indicating that the hemodynamic burden was concentrated in the clinical obesity group (Table 2).

### 3.4. Cross-Classification with Conventional BMI Categories

Cross-classification of standard BMI categories with the Lancet 2025 diagnostic groups showed substantial overlap but also clinically relevant discordances. Although most adults with preclinical or clinical obesity were concentrated in the conventional BMI-defined obesity category, a sizable proportion fell within the overweight range, including 22.9% of those with preclinical obesity and 18.3% of those with clinical obesity. Small but relevant proportions of adults with normal BMI were also classified as having preclinical obesity (2.0%) or clinical obesity (4.9%). Conversely, only 0.6% of participants classified as without obesity according to the Lancet criteria fell within the conventional BMI-obesity range. Standard BMI categories were strongly associated with the Lancet 2025 diagnostic groups (*p* < 0.001); however, BMI alone did not fully capture the clinically meaningful obesity phenotypes identified by the Lancet criteria (Table 3; Figure 2b).

**Table 3:** Distribution of standard body mass index categories across Lancet 2025 obesity categories, Mexican adults aged 20 years and older, ENSANUT 2023 (*n* = 5014).

Standard BMI Category	Without Obesity *N* = 38,620,546	Preclinical Obesity *N* = 24,059,354	Clinical Obesity *N* = 15,235,503	*p*-Value
Underweight	3.8 [3.1–4.6]	0.0 [0.0–0.1]	0.0 [0.0–0.0]	<0.001
Normal weight	46.4 [43.9–49.0]	2.0 [1.4–2.9]	4.9 [4.0–5.9]	
Overweight	49.1 [46.8–51.5]	22.9 [20.5–25.5]	18.3 [15.9–20.8]	
Obesity	0.6 [0.3–1.2]	75.1 [72.5–77.4]	76.9 [74.2–79.3]	

Weighted %, 95% CI, logit method; columns sum to 100%; accounts for the complex survey design. *p*-Value: second-order Rao–Scott chi-square test. Abbreviations: BMI, body mass index; ENSANUT, Encuesta Nacional de Salud y Nutrición.

### 3.5. Qualifying Dysfunction Domains in Clinical Obesity

Among adults classified as having clinical obesity, cardiovascular dysfunction was the most frequent qualifying domain, affecting 78.1% of participants overall, and its frequency increased with higher BMI, from 47.2% in normal-weight individuals to 81.1% in those with BMI-defined obesity.

Metabolic dysfunction was much less common overall (6.7%) but followed a similar gradient, increasing from 0.3% in normal-weight individuals to 8.2% in those with BMI-defined obesity.

Limitations in activities of daily living showed the opposite pattern: identified in 27.6% of participants overall, they were markedly more frequent among adults with normal weight (67.2%) and overweight (48.0%) than among those with BMI-defined obesity (20.0%).

Because the qualifying dysfunction domains were not mutually exclusive, these results indicate that clinical obesity in this population was largely determined by cardiovascular dysfunction, with both cardiovascular and metabolic dysfunction becoming more frequent at higher BMI, whereas functional limitations contributed importantly to cases with lower BMI (Table 4).

**Table 4:** Qualifying dysfunction domains among adults with clinical obesity, by standard body mass index category, ENSANUT 2023 (*n* = 980).

Qualifying Dysfunction Domain	Normal Weight *N* = 739,033	Overweight *N* = 2,781,656	Obesity *N* = 11,714,814	Total *N* = 15,235,503
Metabolic dysfunction	0.3 [0.0–2.3]	2.2 [0.9–5.3]	8.2 [5.9–11.3]	6.7 [4.9–9.2]
Cardiovascular dysfunction	47.2 [35.3–59.5]	73.6 [67.2–79.2]	81.1 [77.6–84.2]	78.1 [75.2–80.8]
Limitations in activities of daily living	67.2 [54.2–77.9]	48.0 [41.9–54.1]	20.0 [16.3–24.2]	27.6 [24.2–31.2]

Weighted % within BMI category, 95% CI, logit method; accounts for the complex survey design. Domains can coexist; columns sum to >100%. Only obesity-attributable domains counted. Abbreviations: BMI, body mass index; ENSANUT, Encuesta Nacional de Salud y Nutrición.

To evaluate whether the low overall prevalence of metabolic dysfunction reflected the conservative definition of its hyperglycemia component, we compared the main analysis with a sensitivity analysis that broadened this component only; the concurrent requirement for hypertriglyceridemia and hypercholesterolemia was unchanged in both analyses. In the main analysis, hyperglycemia was defined as self-reported prediabetes, impaired fasting glucose, or impaired capillary glucose (Section 2.3); the sensitivity analysis additionally counted a self-reported prior diagnosis of type 2 diabetes as meeting this component. Broadening the hyperglycemia component in this way increased the prevalence of clinical obesity from 19.6% (95% CI 18.1–21.1) to 23.3% (21.8–24.9), a difference of 3.78 percentage points with non-overlapping confidence intervals; 248 participants classified as having preclinical obesity in the main analysis met this broader criterion and were reclassified as having clinical obesity (Table S2).

### 3.6. Factors Associated with Lancet 2025 Obesity Categories

In multinomial regression analyses, age was the most consistent factor associated with the Lancet 2025 obesity classification. Across all models, increasing age was associated with higher odds of preclinical and clinical obesity, with stronger effect sizes for clinical obesity. Female sex was associated with higher odds of preclinical obesity across all three models; for clinical obesity, the association strengthened after adjustment for smoking and CESD-7 score (Model 2) but was no longer statistically significant in the fully adjusted sensitivity model (Model 3). Among the behavioral factors, current smoking was independently associated with lower odds of preclinical obesity (but not clinical obesity), and this association persisted after full adjustment. Episodic binge drinking was evaluated but not retained in the final models because of a high proportion of missing data (67%); the CESD-7 score was not independently associated with either obesity category. Physical activity was associated with lower odds of preclinical obesity in the fully adjusted model, although the magnitude was minimal; in the same sensitivity analysis, longer sleep duration was associated with higher odds of clinical obesity (Table 5).

**Table 5:** Complex-sample multinomial logistic regression of factors associated with *The Lancet* 2025 obesity categories, Mexican adults aged 20 years and older, ENSANUT 2023.

Variable	Model 1 OR (95% CI)	Model 2 OR (95% CI)	Model 3 OR (95% CI)
**Preclinical obesity vs. without obesity**			
Age (per year)	1.02 (1.01–1.02) ***	1.01 (1.01–1.02) ***	1.02 (1.02–1.03) ***
Female sex	2.08 (1.71–2.52) ***	2.03 (1.64–2.51) ***	1.74 (1.34–2.26) ***
Current smoking	—	0.78 (0.63–0.97) *	0.74 (0.57–0.95) *
CESD-7 score (per point)	—	1.01 (0.99–1.03)	1.01 (0.98–1.03)
Physical activity (per 1000 METs)	—	—	0.98 (0.97–0.99) **
Sleep duration (per hour)	—	—	0.99 (0.92–1.07)
**Clinical obesity vs. without obesity**			
Age (per year)	1.05 (1.04–1.05) ***	1.05 (1.04–1.05) ***	1.05 (1.04–1.06) ***
Female sex	1.29 (1.06–1.57) *	1.43 (1.15–1.79) **	0.94 (0.72–1.22)
Current smoking	—	1.13 (0.90–1.41)	1.11 (0.87–1.42)
CESD-7 score (per point)	—	1.01 (0.98–1.03)	1.02 (0.99–1.05)
Physical activity (per 1000 METs)	—	—	0.99 (0.97–1.00)
Sleep duration (per hour)	—	—	1.22 (1.11–1.35) ***
Observations (n)	5014	4487	2715

Odds ratios, complex-sample multinomial logistic regression; without obesity as reference. Model 1: age, sex. Model 2: + smoking, CESD-7. Model 3: + physical activity, sleep duration. Definitional variables excluded to avoid circularity. Model 3: complete cases only (sensitivity analysis). * *p* < 0.05; ** *p* < 0.01; *** *p* < 0.001. Abbreviations: CESD-7, seven-item Center for Epidemiologic Studies Depression scale; CI, confidence interval; ENSANUT, Encuesta Nacional de Salud y Nutrición; METs, metabolic equivalents of task; OR, odds ratio.

## 4. Discussion

### 4.1. Principal Findings

To our knowledge, this is one of the first nationally representative studies in Latin America to analyze an operational approximation of *The Lancet* 2025 criteria using population survey data. Applying *The Lancet* 2025 framework to a nationally representative sample of Mexican adults produced a different clinical picture than conventional BMI categories alone.

Preclinical and clinical obesity were both common, and a considerable share of adults with clinical obesity fell within the conventional overweight or normal-weight BMI ranges, indicating that BMI-based screening alone would have missed obesity-related organ dysfunction in this subgroup. Beyond that threshold, BMI plateaued, whereas central adiposity and hemodynamic indicators continued to differ markedly between the two groups.

The distribution of qualifying dysfunction domains in our study also varied by age. Cardiovascular dysfunction predominated among younger adults with clinical obesity, while limitations in activities of daily living became the leading qualifying domain among older adults, and the balance between preclinical and clinical obesity shifted across the life course accordingly.

### 4.2. Comparison with Other Population-Based Studies

One of the most relevant findings of our analysis was that 4.9% of adults with a normal BMI and 18.3% of those with overweight already met the criteria for clinical obesity, a discrepancy consistent with emerging population-based evidence. In Peru, Guerra Valencia et al. [10] found that 13.5% of people with normal BMI and 21% of people with overweight met the criteria for clinical obesity. Individuals classified within lower-risk BMI categories may already have obesity-related dysfunction and therefore go undetected when assessment relies solely on BMI.

A recent analysis of the 2017–2018 National Health and Nutrition Examination Survey (NHANES) reported high concordance between BMI-defined obesity (37.7%) and excess adiposity under the Lancet criteria (37.1%), suggesting that anthropometric confirmation may add limited value at the population level; however, that study did not assess clinical complications or functional limitations and therefore did not address the second step of the Lancet 2025 criteria [7].

A large study of Middle Eastern adults reported a clinical obesity prevalence of 18.8% using ICD-10-based criteria, but without anthropometric confirmation of excess adiposity, limiting comparability; even among individuals with class III obesity, only about one-third met criteria for clinical obesity [9]. Because such estimates depend heavily on the operational definitions and cutoff points applied, they may under- or overestimate the true burden of clinical obesity. The Middle Eastern study, moreover, included hypertension and self-reported diabetes as qualifying dysfunction indicators, a criterion we excluded throughout, in line with the Lancet Commission framework.

Our study revealed a discrepancy between obesity classification based on BMI and that according to *The Lancet*’s criteria. Although clinically relevant, the cross-sectional nature of the study precludes establishing a predictive value for organ dysfunction progression or metabolic disease development. A similar reclassification applied in a Chinese community-based cohort demonstrated that clinical obesity was associated with adverse metabolic, anthropometric, and vascular profiles. However, this analysis, like ours, was cross-sectional. Subsequently, the same research group conducted a 20-year follow-up study (*n* = 2900), which showed that clinical obesity was a significant predictor of incident diabetes, cardiovascular disease, and renal outcomes [11]. The findings suggest that the disparity between BMI and the criteria established by the Lancet is unlikely to be clinically arbitrary.

### 4.3. The Metabolic Domain: Measurement Limitations and Sensitivity Analysis

Among adults with clinical obesity, metabolic dysfunction was the least frequent qualifying domain (6.7%), well below cardiovascular dysfunction. This difference is likely explained in part by how the metabolic domain was operationalized. HDL cholesterol was not measured in ENSANUT 2023, and total cholesterol was substituted for the low-HDL component of the Commission’s criterion (Section 2.3, Table S1). Because total and HDL cholesterol are not interchangeable markers, this substitution probably underestimated the true prevalence of metabolic dysfunction, particularly given that hypoalphalipoproteinemia has been reported to affect 40–60% of Mexican adults [26].

The extent of this underestimation can be partly quantified. In the main analysis, the hyperglycemia component of the metabolic domain was based on self-reported prediabetes, impaired fasting glucose, or impaired capillary glucose; a prior diagnosis of type 2 diabetes was not included, following the Commission’s decision to define metabolic dysfunction through a biochemical cluster rather than a diagnosed disease, since using one disease to define another reduces specificity [7]. When this component was broadened to include a self-reported diagnosis of type 2 diabetes, the prevalence of clinical obesity increased from 19.6% (95% CI 18.1–21.1) to 23.3% (21.8–24.9), a difference of 3.78 percentage points with non-overlapping confidence intervals (Table S2). This gap indicates that the estimated burden of clinical obesity is sensitive to how the metabolic domain is operationalized, and that the main analysis should be interpreted as a conservative lower bound.

Beyond definitional choices, the metabolic domain could be further strengthened using biomarkers not collected in ENSANUT 2023: C-reactive protein and the HOMA-IR index have both been associated with adverse cardiometabolic outcomes in individuals with obesity [27,28], and their inclusion in future survey rounds could allow this domain to be assessed independently of self-reported diagnoses.

### 4.4. Age-Related Phenotypic Shifts

The distribution of Lancet 2025 obesity categories differed markedly by age (Table S3): the preclinical-to-clinical ratio and the prevalence of cardiovascular dysfunction both peaked among adults aged 30–39 years (2.53 and 97.3%, respectively) and declined thereafter to 0.30 and 46.7%, respectively, among those aged 70 years and older, with the ratio falling below 1 from the 60–69-year group onward. Activity limitations rose over the same range, from 5.8% to 70.2%, while metabolic dysfunction remained low and inconsistent throughout (1.9–13.6%).

This shift admits two explanations that cannot be disentangled with the present data. Biologically, age-related adipose tissue remodeling (including reduced adipogenesis, fibrosis, and chronic low-grade inflammation) [29] plausibly favors a transition from a cardiovascular to a functional phenotype. Methodologically, the Katz and Lawton–Brody instruments were administered primarily to older participants (over 83% of recipients were aged 60 years or older), so functional impairment among younger adults was detected only sporadically, regardless of its presence. Diagnostic performance is therefore not uniform across cohorts: comparisons are directly interpretable for age-independent domains such as cardiovascular dysfunction but partly reflect differential ascertainment for daily activities.

Two age-relevant phenotypes could not be evaluated in ENSANUT 2023: sarcopenic obesity and metabolic dysfunction-associated steatotic liver disease (MASLD) [30]. ENSANUT lacks the muscle-function and complete battery measures required by the ESPEN–EASO algorithm [31] and includes no hepatic imaging or biochemistry. Both phenotypes nonetheless map onto the framework: the Commission counts steatotic liver disease as a criterion only when accompanied by fibrosis [7], so steatosis alone would be classified as preclinical rather than clinical obesity, while sarcopenia is not itself a criterion but qualifies through limitations of mobility or daily activities [7] (the same domain that predominated among older adults in this cohort). The ESPEN–EASO stage I/II distinction mirrors this preclinical/clinical structure [31], and because BMI underestimates fat mass in individuals who have lost muscle mass, sarcopenic obesity is the phenotype least reliably captured by BMI; its systematic assessment would likely further widen the discordance reported here.

### 4.5. Correlates of Obesity Category

Three nested models tested the stability of factors associated with Lancet 2025 obesity categories: Model 1 (age, sex), Model 2 (adding smoking and depressive symptoms), and Model 3, restricted to participants with complete covariate data, including physical activity and sleep duration (*n* = 2715, 54% of the sample; Table 5). Differences across models may reflect effect modification by the added covariates, or selective participation in this smaller subsample.

Age was the most consistent correlate, with virtually unchanged odds ratios across all three models for preclinical (OR approximately 1.02/year) and clinical obesity (OR approximately 1.05/year) (the latter consistent with the stronger age-related qualifying-domain shift described above), supporting a confident interpretation independent of the covariates added later.

Female sex showed a more complex pattern: significant for preclinical obesity across all models (OR 2.08 to 1.74) but shifting from a positive association with clinical obesity in Models 1–2 (OR up to 1.43) to a null point estimate in Model 3 (OR 0.94), a change in the estimate itself, not just precision. This is not fully explained by power loss: either the complete-case subsample differs from the full sample in sex-patterned ways, or physical activity and sleep duration, added only in Model 3, partly account for the earlier association. A sex difference in dysfunction risk remains biologically plausible given the more android, visceral fat distribution typical of men, which is more strongly linked to inflammation, insulin resistance, and cardiometabolic risk [32]. However, which explanation applies cannot be determined from the present study.

Current smoking was independently associated with lower odds of preclinical obesity (OR 0.78–0.74 across Models 2–3) but not clinical obesity. A likely biological pathway is nicotine-induced activation of hypothalamic α3β4 nicotinic receptors on POMC neurons, which engages the melanocortin pathway that suppresses appetite [33,34]. Because this mechanism acts on adiposity accumulation rather than on cardiovascular or metabolic pathways, it may lower the odds of preclinical obesity without protecting against dysfunction once present.

Longer sleep duration was associated with higher odds of clinical obesity in Model 3 (OR 1.22/hour; 95% CI 1.11–1.35), which was the only model testing this variable. Despite the narrow CI, this should be treated as preliminary because it was assessed once, in a smaller subsample, and the cross-sectional design cannot rule out reverse causation or confounding (e.g., comorbidity-related sleep changes in older adults). The direction is consistent with a U-shaped sleep-obesity association, though the long-sleep arm has generally been reported as weaker than ours [35], warranting replication.

### 4.6. Clinical and Public Health Implications

Susceptibility to obesity-related dysfunction varies substantially among individuals with similar adiposity [36], and BMI, waist circumference, and waist-to-hip ratio have each shown limited added value for cardiovascular risk prediction once major clinical risk factors are accounted for [37]. Consistent with this, our findings show that BMI alone provides little further information once the obesity threshold is reached. The practical implication is that a BMI ≥ 30 kg/m^2^ should not, by itself, be assumed to indicate preclinical rather than clinical obesity; a second, low-cost screening step, such as blood pressure and waist-to-height ratio, is needed before that assumption is made. Direct quantification of body fat with DXA or bioimpedance would refine this distinction further but remains limited by cost and portability for routine clinical use or population-based surveys [38,39]. Waist-to-height ratio, which has widely validated cutoff points by region, sex, and age [21] and is already incorporated into the present classification, therefore remains a practical and defensible confirmatory step for large-scale surveys such as ENSANUT.

Beyond screening, this framework has implications for how obesity is understood clinically. Despite being a chronic, multifactorial disease comparable to asthma, diabetes, or cancer, obesity has traditionally been treated as an individual responsibility attributable to lifestyle and diet, a framing with documented consequences for stigma: individuals with overweight or obesity often delay seeking care because they attribute weight loss solely to personal responsibility and associate excess weight with a lack of self-discipline [40,41]. By requiring evidence of dysfunction rather than body size alone, *The Lancet* 2025 criteria apply the same diagnostic logic already used for diabetes or cancer, in which diagnosis does not depend on BMI category. This shift, from an almost exclusively anthropometric diagnosis to one requiring a clinical picture or biomarker, could help reduce stigma for patients and clinicians alike.

This reclassification is unlikely to drive overmedicalization. Current evidence on weight stigma points to the opposite pattern in clinical practice: even among patients who already show metabolic dysfunction attributable to excess adiposity, clinicians tend to favor primary prevention over pharmacological or surgical treatment [40]. In the United States, fewer than 2% of patients with overweight or obesity have ever been prescribed an obesity medication [42], and a systematic review found that fewer than 1% of candidates for bariatric surgery are referred for the procedure [43]. Rather than expanding treatment, a preclinical/clinical classification could instead serve as a marker for timely referral, helping to counteract this pattern of delayed intervention.

Two practical limitations of *The Lancet* 2025 approach itself should be acknowledged. First, distinguishing preclinical from clinical obesity requires evaluating multiple organ systems (cardiovascular, renal, metabolic, respiratory, musculoskeletal), which demands laboratory testing, imaging, and specialist evaluation beyond what routine clinical care or population surveys typically provide; the present study itself could only approximate a minority of these criteria, a limitation discussed further below. Second, because domain ascertainment depends on what each health system or survey measures, comparing prevalence and clinical characteristics across studies remains difficult, as illustrated by the divergence, noted above, between our estimates and those of other cohorts applying the same framework with different instruments. Addressing both limitations will require converging on a minimum, feasible set of criteria that balances diagnostic completeness against practicality for routine use.

### 4.7. Strengths and Limitations

This study has several methodological strengths. First, it draws on a complex, nationally representative sampling design, allowing estimates that generalize to the adult Mexican population rather than to a convenience sample. Second, to our knowledge, it is among the first studies in Latin America to apply the complete two-step Lancet 2025 framework using measured signs of organ dysfunction rather than relying solely on recorded diagnoses. Third, the operationalization of each diagnostic criterion is described in detail (Table S1), allowing other research groups to reproduce or adapt this classification in surveys with different variable availability. Fourth, multiple sensitivity analyses (Table S2; Model 3, a complete-case regression) allowed the robustness of the main findings to specific analytic choices to be quantified rather than only acknowledged.

This study also has several limitations, which should be interpreted alongside its strengths. Of the eighteen diagnostic criteria defined by the Commission for adults, only four could be evaluated in ENSANUT 2023: raised arterial blood pressure was ascertained in full, while the metabolic, renal, and functional domains were approximated using proxy indicators; the remaining fourteen criteria, including hepatic, respiratory, reproductive, musculoskeletal, and lymphatic dysfunction, had no corresponding indicator and could not be evaluated in any participant (Table S1). In addition, some modules were administered only to specific subgroups (for example, the Katz and Lawton–Brody instruments for activities of daily living, administered predominantly to adults aged 60 years and older, as noted above) so that 199 participants (4.0%) could not be assessed for any domain.

This incomplete ascertainment does not bias the classification randomly. Clinical obesity requires only a single positive criterion, whereas preclinical obesity requires the absence of dysfunction across all assessable domains; a domain that could not be assessed can therefore only move a participant from clinical toward preclinical obesity, never the reverse. Our estimate of clinical obesity should consequently be interpreted as a lower bound, and that of preclinical obesity as an upper bound. This effect can be partly bound for the subset of participants with confirmed excess adiposity in whom no domain could be assessed: had all 90 such participants met a dysfunction criterion, the prevalence of clinical obesity would rise from 19.6% to 21.0%, and that of preclinical obesity would fall from 30.9% to 29.4%. The impact of the fourteen criteria that could not be evaluated in any participant cannot be bounded from these data, so the true prevalence of clinical obesity likely exceeds our estimate by more than this margin.

A related limitation concerns the relative contribution of the cardiovascular domain. Because elevated blood pressure (≥120/80 mmHg) is a comparatively broad, fully ascertained criterion, whereas other domains were approximated with narrower proxy indicators, cardiovascular dysfunction may be over-represented relative to domains that were harder to capture. Because the qualifying domains were not mutually exclusive, a high frequency of cardiovascular dysfunction does not preclude the coexistence of metabolic dysfunction or functional limitations in the same participants (Table 4).

The metabolic domain has a further limitation, noted above: HDL cholesterol was not measured in ENSANUT 2023, and its substitution with total cholesterol likely underestimated the prevalence of metabolic dysfunction, particularly given that hypoalphalipoproteinemia has been reported to affect 40–60% of Mexican adults [26].

The cross-sectional design of ENSANUT 2023 also precludes causal inference about the relationships among adiposity, organ dysfunction, and functional limitation.

Finally, because the analytic sample was derived from linked ENSANUT modules and was constrained by the availability of complete data across them, some degree of selection bias cannot be ruled out.

## 5. Conclusions

BMI alone is insufficient to capture clinically meaningful obesity phenotypes. Incorporating central adiposity measures and assessment of obesity-related dysfunction into obesity surveillance, rather than relying on BMI alone, could improve population-level risk stratification. Future studies should extend these findings through longitudinal designs, the inclusion of biochemical markers of insulin resistance and inflammation, and more granular body-composition assessments to refine how *The Lancet* 2025 criteria are operationalized. Distinguishing excess body weight, an anthropometric finding, from obesity, a condition with clinical consequences, matters for prioritizing treatment, allocating resources, and countering simplistic interpretations of body size that reinforce weight-related stigma.

## Data Availability

The data analyzed in this study are publicly available in the official ENSANUT repository of the National Institute of Public Health (INSP) at https://ensanut.insp.mx/ (accessed on 19 February 2025). No new data were generated.

## Supplementary Materials

The following supporting information can be downloaded at: https://www.mdpi.com/article/10.3390/nu18152582/s1. Table S1: Operationalization of *The Lancet Diabetes & Endocrinology* Commission (2025) diagnostic criteria for clinical obesity using ENSANUT 2023 (*n* = 5014 adults aged ≥20 years); Table S2: Sensitivity of the prevalence of preclinical and clinical obesity to the definition of the metabolic dysfunction domain, Mexican adults aged 20 years and older, ENSANUT 2023 (*n* = 5014); Table S3: Prevalence of preclinical and clinical obesity and distribution of qualifying dysfunction domains by age group, Mexican adults aged 20 years and older, ENSANUT 2023 (*n* = 5014).

## Abbreviation

BMI	Body mass index
WHtR	Waist-to-height ratio
WC	Waist circumference
SBP	Systolic blood pressure
DBP	Diastolic blood pressure
CI	Confidence interval
SE	Standard error
OR	Odds ratio
ENSANUT	Encuesta Nacional de Salud y Nutrición (National Health and Nutrition Survey)
IPAQ	International Physical Activity Questionnaire
METs	Metabolic equivalents
CESD-7	Seven-Item Center for Epidemiologic Studies Depression Scale
HDL	High-density lipoprotein
ICD-10	International Classification of Diseases, 10th Revision
NHANES	National Health and Nutrition Examination Survey
SARC-F	Strength, Assistance with walking, Rise from a chair, Climb stairs, and Falls questionnaire
WHO	World Health Organization
BP	Blood pressure
CV	Cardiovascular
INSP	Instituto Nacional de Salud Pública

## References

[1] Phelps N.H., Singleton R.K., Zhou B., Heap R.A., Mishra A., Bennett J.E., Paciorek C.J., Lhoste V.P., Carrillo-Larco R.M., Stevens G.A. (2024). Worldwide Trends in Underweight and Obesity from 1990 to 2022: A Pooled Analysis of 3663 Population-Representative Studies with 222 Million Children, Adolescents, and Adults. Lancet.

[2] World Health Organization (2026). Obesity and Overweight. https://www.who.int/news-room/fact-sheets/detail/obesity-and-overweight.

[3] World Obesity Federation (2025). World Obesity Atlas 2025.

[4] Kerr J.A., Patton G.C., Cini K.I., Abate Y.H., Abbas N., Abd Al Magied A.H.A., Abd ElHafeez S., Abd-Elsalam S., Abdollahi A., Abdoun M. (2025). Global, Regional, and National Prevalence of Child and Adolescent Overweight and Obesity, 1990–2021, with Forecasts to 2050: A Forecasting Study for the Global Burden of Disease Study 2021. Lancet.

[5] Wu Y., Li D., Vermund S.H. (2024). Advantages and Limitations of the Body Mass Index (BMI) to Assess Adult Obesity. Int. J. Environ. Res. Public Health.

[6] Krakauer N.Y., Krakauer J.C. (2025). Novel Anthropometric Indices: An Allometric Perspective. Endocrines.

[7] Rubino F., Cummings D.E., Eckel R.H., Cohen R.V., Wilding J.P.H., Brown W.A., Stanford F.C., Batterham R.L., Farooqi I.S., Farpour-Lambert N.J. (2025). Definition and Diagnostic Criteria of Clinical Obesity. Lancet Diabetes Endocrinol..

[8] Aryee E.K., Zhang S., Selvin E., Fang M. (2025). Prevalence of Obesity with and Without Confirmation of Excess Adiposity Among US Adults. JAMA.

[9] Abdelgadir E., Rashid F., Bashier A., Zidan M., McGowan B., Alawadi F. (2025). Prevalence of Overweight and Obesity in Adults from the Middle East: A Large-Scale Population-Based Study. Diabetes Obes. Metab..

[10] Guerra Valencia J., Hernández-Vásquez A., Mayta-Tristán P., Saavedra-Garcia L., Vargas-Fernández R. (2025). The New Definition of Obesity: An Analysis of a Population-Based Survey in an Andean Country. Lancet Reg. Health Am..

[11] Lui D.T.W., Fong C.H.Y., Zou X., Xu A., Tse H.F., Woo J., Lam T.H., Woo Y.C., Cheung B.M.Y., Janus E. (2026). The Impact of the Lancet Commission Definition of Obesity on Its Prevalence and Implications on Long-Term Cardiovascular-Kidney-Metabolic Outcomes in East Asians: Observational Study of Two Community-Based Cohorts. PLoS Med..

[12] Campos-Nonato I., Galván-Valencia O., Hernández-Barrera L., Oviedo-Solís C., Barquera S. (2023). Prevalence of Obesity and Associated Risk Factors in Mexican Adults: Results of the ENSANUT 2022. Salud Publica Mex..

[13] Shamah-Levy T., Lazcano-Ponce E., Cuevas-Nasu L., Romero-Martínez M., Gaona-Pineda E., Gómez-Acosta L., Mendoza-Alvarado L., Méndez-Gómez-Humarán I. (2024). Encuesta Nacional de Salud y Nutrición Continua 2023. Resultados Nacionales.

[14] Cuschieri S., Zhang N., Grech E., Grech S. (2026). Comparing Anthropometric Measures and Lancet Commission Definitions in Relation to Hypertension: Population-Level Evidence from a Small Island with High Metabolic Burden. Public Health Pract..

[15] Chaudhary P., Yang S., Waldrop S., Katzmarzyk P.T., Staiano A.E. (2026). Prevalence of Preclinical and Clinical Obesity Among US Children and Adolescents Aged 5 to 18 Years: NHANES 2017–2023. Obesity.

[16] Verma M., Halder P., Aditi A., Kapoor N., Esht V., Ahamed W.M., Kakkar R., Kalra S. (2026). Novel Clinical and Pre-Clinical Obesity Estimates in Older Indian Adults: Insights from a Nationally Representative Dataset. Diabetes Ther..

[17] Wang Z., Du W., Wei X., Li S., Zhang J., Ju L., Gong W., Guo Q., Xu X., Cheng X. (2026). Clinical Obesity Among Chinese Adults: Prevalence, Multimorbidity Burden, and Associations with Physical Activity. Nutrients.

[18] Martín O., Hernández R.A. (2013). Ecuaciones de Predicción del Peso Corporal para Adultos Venezolanos. Antropo.

[19] WHO Expert Consultation (2004). Appropriate Body-Mass Index for Asian Populations and Its Implications for Policy and Intervention Strategies. Lancet.

[20] Asociación Argentina de Dietistas y Nutricionistas (AADYND) (2023). Valoración del Estado Nutricional de Personas Mayores.

[21] Rangel-Baltazar E., Rodríguez-Ramírez S., Cuevas-Nasu L., Shamah-Levy T., Méndez-Gómez-Humarán I., Rivera J.A. (2023). Short Stature Modifies the Waist-to-Height Ratio Cut-off Points as an Indicator of Cardiovascular Risk in Mexican Adult Women and Men. Arch. Med. Res..

[22] Merati M., Mohebi F., Alipour E., Masinaei M., Pooyan A., Mehdipour P., Mohajer B., Komaki H., Mobarakabadi M., Farzadfar F. (2024). Validation of the Self-Reported Diagnosis of Diabetes Mellitus, Hypercholesterolemia, and Hypertension in Iran; STEPS 2016. J. Diabetes Metab. Disord..

[23] Jones D.W., Ferdinand K.C., Taler S.J., Johnson H.M., Shimbo D., Abdalla M., Altieri M.M., Bress A.P., Carter J., Commodore-Mensah Y. (2025). 2025 AHA/ACC/AANP/AAPA/ABC/ACCP/ACPM/AGS/AMA/ASPC/NMA/PCNA/SGIM Guideline for the Prevention, Detection, Evaluation and Management of High Blood Pressure in Adults: A Report of the American College of Cardiology/American Heart Association Joint Committee on Clinical Practice Guidelines. Circulation.

[24] Salinas-Rodríguez A., Manrique-Espinoza B., De la Cruz-Góngora V., Rivera-Almaraz A. (2019). Socioeconomic Inequalities in Health and Nutrition among Older Adults in Mexico. Salud Publica Mex..

[25] Rathnayake N., Abeygunasekara T., Liyanage G., Subasinghe S., De Zoysa W., Palangasinghe D., Lekamwasam S. (2025). SARC-F: An Effective Screening Tool for Detecting Sarcopenia and Predicting Health-Related Quality of Life in Older Women in Sri Lanka. BMC Geriatr..

[26] Flores-Luna L., Rojas-Martínez R., Escamilla-Núñez C., Castro-Porras L.V., Hernández Cadena L., Romero-Martínez M., Aguilar-Salinas C.A. (2025). Prevalencia de Factores de Riesgo Cardiometabólico en México, ENSANUT 2021. Salud Publica Mex..

[27] González-González J.G., Violante-Cumpa J.R., Zambrano-Lucio M., Burciaga-Jimenez E., Castillo-Morales P.L., Garcia-Campa M., Solis R.C., González-Colmenero A.D., Rodríguez-Gutiérrez R. (2022). HOMA-IR as a Predictor of Health Outcomes in Patients with Metabolic Risk Factors: A Systematic Review and Meta-Analysis. High Blood Press. Cardiovasc. Prev..

[28] Powell-Wiley T.M., Poirier P., Burke L.E., Després J.P., Gordon-Larsen P., Lavie C.J., Lear S.A., Ndumele C.E., Neeland I.J., Sanders P. (2021). Obesity and Cardiovascular Disease: A Scientific Statement from the American Heart Association. Circulation.

[29] Von Bank H., Kirsh C., Simcox J. (2021). Aging Adipose: Depot Location Dictates Age-Associated Expansion and Dysfunction. Ageing Res. Rev..

[30] Rinella M.E., Lazarus J.V., Ratziu V., Francque S.M., Sanyal A.J., Kanwal F., Romero D., Abdelmalek M.F., Anstee Q.M., Arab J.P. (2023). A Multisociety Delphi Consensus Statement on New Fatty Liver Disease Nomenclature. J. Hepatol..

[31] Donini L.M., Busetto L., Bischoff S.C., Cederholm T., Ballesteros-Pomar M.D., Batsis J.A., Bauer J.M., Boirie Y., Cruz-Jentoft A.J., Dicker D. (2022). Definition and Diagnostic Criteria for Sarcopenic Obesity: ESPEN and EASO Consensus Statement. Clin. Nutr..

[32] Chang E., Varghese M., Singer K. (2018). Gender and Sex Differences in Adipose Tissue. Curr. Diab. Rep..

[33] Mineur Y.S., Abizaid A., Rao Y., Salas R., DiLeone R.J., Gündisch D., Diano S., De Biasi M., Horvath T.L., Gao X.-B. (2011). Nicotine Decreases Food Intake Through Activation of POMC Neurons. Science.

[34] Hu T., Yang Z., Li M.D. (2018). Pharmacological Effects and Regulatory Mechanisms of Tobacco Smoking Effects on Food Intake and Weight Control. J. Neuroimmune Pharmacol..

[35] Che T., Yan C., Tian D., Zhang X., Liu X., Wu Z. (2021). The Association Between Sleep and Metabolic Syndrome: A Systematic Review and Meta-Analysis. Front. Endocrinol..

[36] Kim M., Yoo H., Ko J., Lee J. (2020). Metabolically Unhealthy Overweight Individuals Have High Lysophosphatide Levels, Phospholipase Activity, and Oxidative Stress. Clin. Nutr..

[37] (2011). Emerging Risk Factors Collaboration. Separate and Combined Associations of Body-Mass Index and Abdominal Adiposity with Cardiovascular Disease: Collaborative Analysis of 58 Prospective Studies. Lancet.

[38] Marra M., Sammarco R., De Lorenzo A., Iellamo F., Siervo M., Pietrobelli A., Donini L.M., Santarpia L., Cataldi M., Pasanisi F. (2019). Assessment of Body Composition in Health and Disease Using Bioelectrical Impedance Analysis (BIA) and Dual Energy X-Ray Absorptiometry (DXA): A Critical Overview. Contrast Media Mol. Imaging.

[39] Silveira E.A., Castro M.C.R., Rezende A.T.O., dos Santos Rodrigues A.P., Delpino F.M., Oliveira E.S., Corgosinho F.C., de Oliveira C. (2024). Body Composition Assessment in Individuals with Class II/III Obesity: A Narrative Review. BMC Nutr..

[40] Talumaa B., Brown A., Batterham R.L., Kalea A.Z. (2022). Effective Strategies in Ending Weight Stigma in Healthcare. Obes. Rev..

[41] Robinson K.M., Scherer A.M., Zorn A.N., Mengeling M.A., Laroche H.H. (2025). Association Between Weight Stigma Experiences in Healthcare and Self-Reported Healthcare Avoidance in a National Sample. Obes. Sci. Pract..

[42] Semla T.P., Ruser C., Good C.B., Yanovski S.Z., Ames D., Copeland L.A., Billington C., Ferguson U.I., Aronne L.J., Wadden T.A. (2017). Pharmacotherapy for Weight Management in the VHA. J. Gen. Intern. Med..

[43] Funk L.M., Jolles S., Fischer L.E., Voils C.I. (2015). Patient and Referring Practitioner Characteristics Associated with the Likelihood of Undergoing Bariatric Surgery: A Systematic Review. JAMA Surg..

