# Supplementary material for: Operational Approach to the Lancet 2025 Obesity Framework in Mexican Adults: Prevalence and Clinical Characteristics
Source: Nutrients. 2026 Aug 6;18(15):2582. doi: 10.3390/nu18152582 (PMC13467961; doi:10.3390/nu18152582)
Supplement: Supplementary file 1 [file nutrients-18-02582-s001.zip › Table_S1.pdf]

Table S1. Operationalization of the Lancet Diabetes & Endocrinology Commission (2025) diagnostic criteria for clinical obesity using ENSANUT 2023 (n = 5014 adults aged ≥ 20 years).

Organ or Tissue System	Lancet 2025 Diagnostic Criterion (Grade of Consensus)	Operational Definition Applied	Ascertainment	Participants Evaluable, n (%)	Contributing to Clinical Obesity, n
**Step 1. Confirmation of Excess Adiposity (Anthropometric Component)**					
Anthropometry	Direct body fat measurement, e.g., DXA or bioimpedance (A, 98%)	No corresponding indicator available in ENSANUT 2023	Not ascertainable	0 (0.0)	—
Anthropometry	At least one anthropometric criterion in addition to BMI (A, 98%)	BMI ≥ 30 kg/m² plus one altered measure of central adiposity (waist circumference or waist-to-height ratio)	Full	5014 (100.0)	—
Anthropometry	At least two anthropometric criteria regardless of BMI (A, 98%)	Two altered measures of central adiposity irrespective of BMI	Full	5014 (100.0)	—
Anthropometry	Excess adiposity assumed at substantially high BMI (A, 98%)	BMI ≥ 40 kg/m² alone; this criterion confirmed adiposity only and did not by itself establish clinical obesity	Full	5014 (100.0)	—
**Step 2. Assessment of Obesity-Related Organ Dysfunction or Functional Limitation (Clinical Component)**					
Central nervous system	Signs of raised intracranial pressure such as vision loss and/or recurrent headaches (A, 93%)	No corresponding indicator available in ENSANUT 2023	Not ascertainable	0 (0.0)	0
Upper airways	Apnoeas or hypopnoeas during sleep due to increased upper airways resistance (U, 100%)	No corresponding indicator available in ENSANUT 2023	Not ascertainable	0 (0.0)	0
Respiratory	Hypoventilation, breathlessness and/or wheezing due to reduced lung or diaphragmatic compliance (U, 100%)	No corresponding indicator available in ENSANUT 2023	Not ascertainable	0 (0.0)	0
Cardiovascular (arterial)	Raised arterial blood pressure (U, 100%)	Measured systolic > 120 mmHg or diastolic > 80 mmHg (mean of three readings, available in 3,155 participants), or previously diagnosed hypertension	Full	4809 (95.9)	656
Cardiovascular (ventricular)	Reduced left ventricular systolic function — HFrEF (A, 96%)	No corresponding indicator available in ENSANUT 2023	Not ascertainable	0 (0.0)	0
Cardiovascular (atrial)	Chronic or recurrent atrial fibrillation (A, 98%)	No corresponding indicator available in ENSANUT 2023	Not ascertainable	0 (0.0)	0
Cardiovascular (pulmonary)	Pulmonary artery hypertension (A, 96%)	No corresponding indicator available in ENSANUT 2023	Not ascertainable	0 (0.0)	0
Cardiovascular (diastolic)	Chronic fatigue and lower limb oedema due to impaired diastolic function — HFpEF (U, 100%)	No corresponding indicator available in ENSANUT 2023	Not ascertainable	0 (0.0)	0
Cardiovascular (thrombosis)	Recurrent deep vein thrombosis and/or pulmonary thromboembolic disease (A, 90%)	No corresponding indicator available in ENSANUT 2023	Not ascertainable	0 (0.0)	0
Metabolism	Cluster of hyperglycemia, high triglyceride levels and low HDL cholesterol (U, 100%)	Hyperglycemia together with altered lipid indicators; total cholesterol was used in place of HDL cholesterol, which was not available	Partial	4491 (89.6)	64
Liver	Metabolic dysfunction-associated steatotic liver disease with hepatic fibrosis (U, 100%)	No corresponding indicator available in ENSANUT 2023	Not ascertainable	0 (0.0)	0

Organ or Tissue System	Lancet 2025 Diagnostic Criterion (Grade of Consensus)	Operational Definition Applied	Ascertainment	Participants Evaluable, *n* (%)	Contributing to Clinical Obesity, *n*
Renal	Microalbuminuria with reduced eGFR (A, 96%)	Self-reported physician diagnosis of kidney disease; neither albuminuria nor estimated glomerular filtration rate was available. Not incorporated into the classification	Partial	4491 (89.6)	0
Urinary	Recurrent or chronic urinary incontinence (U, 100%)	No corresponding indicator available in ENSANUT 2023	Not ascertainable	0 (0.0)	0
Reproductive (female)	Anovulation, oligomenorrhoea and polycystic ovary syndrome (U, 100%)	No corresponding indicator available in ENSANUT 2023	Not ascertainable	0 (0.0)	0
Reproductive (male)	Male hypogonadism (A, 96%)	No corresponding indicator available in ENSANUT 2023	Not ascertainable	0 (0.0)	0
Musculoskeletal	Chronic, severe knee or hip pain with joint stiffness and reduced range of motion (U, 100%)	A variable for this domain was defined in the working dataset but contained no valid observations	Not ascertainable	0 (0.0)	0
Lymphatic	Lower limb lymphoedema causing chronic pain and/or reduced range of motion (A, 98%)	No corresponding indicator available in ENSANUT 2023	Not ascertainable	0 (0.0)	0
Limitations of day-to-day activities	Significant, age-adjusted limitations of mobility or other basic activities of daily living (U, 100%)	Dependence on the Katz index of activities of daily living or on the Lawton–Brody instrumental scale	Partial	4497 (89.7)	339

[a] Grades of consensus reproduced from the Commission [7] (U = 100% agreement; A = 90–99%). Percentages are unweighted (analytic sample, *n* = 5014). [b] Full: assessed with directly measured variables meeting the Commission definition. Partial: approximated with self-reported or proxy indicators; positive findings are informative, negative findings do not exclude dysfunction. Not ascertainable: no corresponding indicator available. [c] Because clinical obesity requires only one positive criterion while preclinical obesity requires absence of dysfunction across all assessable domains, partial or non-ascertainable criteria can only under-ascertain clinical obesity (Section 2.3); reported prevalence is therefore a conservative lower bound. [d] Of 2676 participants with confirmed excess adiposity, 980 met ≥1 dysfunction criterion (clinical obesity; weighted *N* = 15,235,503) and 1696 did not (preclinical obesity; weighted *N* = 23,059,354); domains are not mutually exclusive. Abbreviations: BMI, body mass index; DXA, dual-energy X-ray absorptiometry; eGFR, estimated glomerular filtration rate; ENSANUT, Encuesta Nacional de Salud y Nutrición; HDL, high-density lipoprotein; HFpEF, heart failure with preserved ejection fraction; HFrEF, heart failure with reduced ejection fraction.
