# Supplementary material for: Operational Approach to the Lancet 2025 Obesity Framework in Mexican Adults: Prevalence and Clinical Characteristics
Source: Nutrients. 2026 Aug 6;18(15):2582. doi: 10.3390/nu18152582 (PMC13467961; doi:10.3390/nu18152582)
Supplement: Supplementary file 1 [file nutrients-18-02582-s001.zip › Table_S2.pdf]

Table S2. Sensitivity of the prevalence of preclinical and clinical obesity to the definition of the metabolic dysfunction domain, Mexican adults aged 20 years and older, ENSANUT 2023 (n = 5,014).

Obesity category	Participants, n	Weighted population, N	Prevalence, % (95% CI)	Difference, pp
**Main analysis: metabolic dysfunction restricted to self-reported hyperglycemia**				
Without obesity	2338	38,620,546	49.6 (47.8–51.4)	Reference
Preclinical obesity	1696	24,059,354	30.9 (29.2–32.6)	Reference
Clinical obesity	980	15,235,503	19.6 (18.1–21.1)	Reference
**Sensitivity analysis: metabolic dysfunction extended to self-reported type 2 diabetes**				
Without obesity	2338	38,620,546	49.6 (47.8–51.4)	+0.00
Preclinical obesity	1448	21,116,864	27.1 (25.5–28.8)	−3.78
Clinical obesity	1228	18,177,992	23.3 (21.8–24.9)	+3.78

Weighted to the national population; Main analysis: metabolic dysfunction based on self-reported hyperglycemia (Section 2.3). Sensitivity analysis: additionally included self-reported type 2 diabetes diagnosis. Abbreviations: CI, confidence interval; ENSANUT, Encuesta Nacional de Salud y Nutrición; pp, percentage points.
