# Supplementary material for: Operational Approach to the Lancet 2025 Obesity Framework in Mexican Adults: Prevalence and Clinical Characteristics
Source: Nutrients. 2026 Aug 6;18(15):2582. doi: 10.3390/nu18152582 (PMC13467961; doi:10.3390/nu18152582)
Supplement: Supplementary file 1 [file nutrients-18-02582-s001.zip › Table_S3.pdf]

Table S3. Prevalence of preclinical and clinical obesity and distribution of qualifying dysfunction domains by age group, Mexican adults aged 20 years and older, ENSANUT 2023 (n = 5014).

Age group, years	n	Weighted N	Without obesity, % (95% CI)	Preclinical obesity, % (95% CI)	Clinical obesity, % (95% CI)	Preclinical-to-clinical ratio	Cardiovascular, %	Metabolic, %	Daily activities, %
20–29	918	17,877,447	72.0 (69.0–74.9)	19.5 (16.8–22.5)	8.5 (7.2–10.1)	2.28	94.7	5.6	5.8
30–39	895	17,702,974	50.8 (46.3–55.2)	35.3 (31.3–39.5)	14.0 (11.4–17.0)	2.53	97.3	1.9	9.0
40–49	1074	15,371,639	41.5 (36.2–47.0)	39.6 (35.3–43.9)	18.9 (15.7–22.6)	2.09	93.9	10.0	7.3
50–59	957	12,434,135	34.4 (30.6–38.4)	42.6 (38.9–46.3)	23.0 (20.3–25.9)	1.85	88.4	13.6	0.2
60–69	723	8,981,716	40.4 (35.7–45.3)	25.1 (21.7–28.8)	34.5 (30.6–38.6)	0.73	54.4	4.5	60.9
≥70	447	5,547,492	44.5 (39.8–49.4)	12.7 (10.0–16.0)	42.8 (38.3–47.4)	0.30	46.7	4.0	70.2

Weighted; 95% CI by the logit method with Taylor series linearization, within each age stratum. Categories sum to 100% within stratum. Last three columns: % of clinical obesity qualifying through each domain (domains coexist, sum to >100%). Abbreviations: CI, confidence interval; ENSANUT, Encuesta Nacional de Salud y Nutrición.
